# Supplementary material for: Impact of Age-Related Genetic Differences on the Therapeutic Outcome of Papillary Thyroid Cancer
Source: Cancers (Basel). 2020 Feb 14;12(2):448. doi: 10.3390/cancers12020448 (PMC7072359; doi:10.3390/cancers12020448)
Supplement: Supplementary file 1 [file cancers-12-00448-s001.pdf]

Uncropped images of the immunoblot analysis.

Supplementary Figure 1B: Immunoblot analysis with antibody- related to marker of the FGFR and EMT on the GSPY and GSPO cells. Equal amounts of protein (20 μg) were separated on 8–10% sodium dodecyl sulfate-polyacrylamide gels.

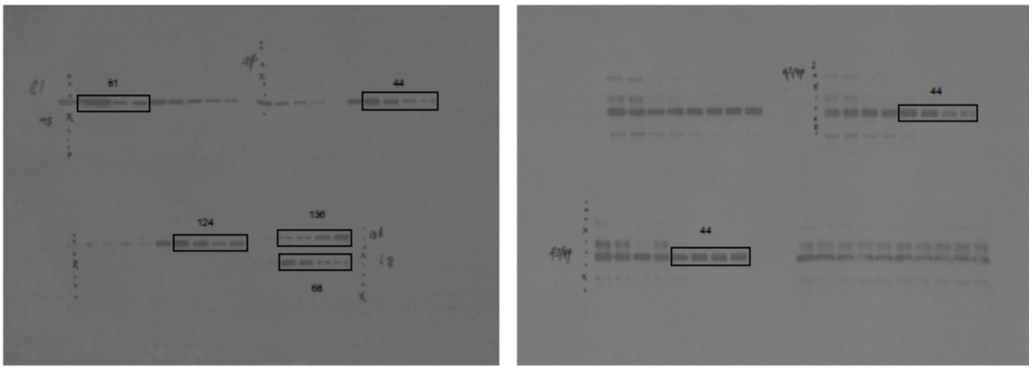

Supplementary Figure 3A: Immunoblot analysis with antibody- related to marker of the FGFR and EMT on the GSPY and GSPO cells after treatment of a multikinase inhibitor, sorafenib and lenvatinib. Equal amounts of protein (20 μg) were separated on 8–10% sodium dodecyl sulfate-polyacrylamide gels.

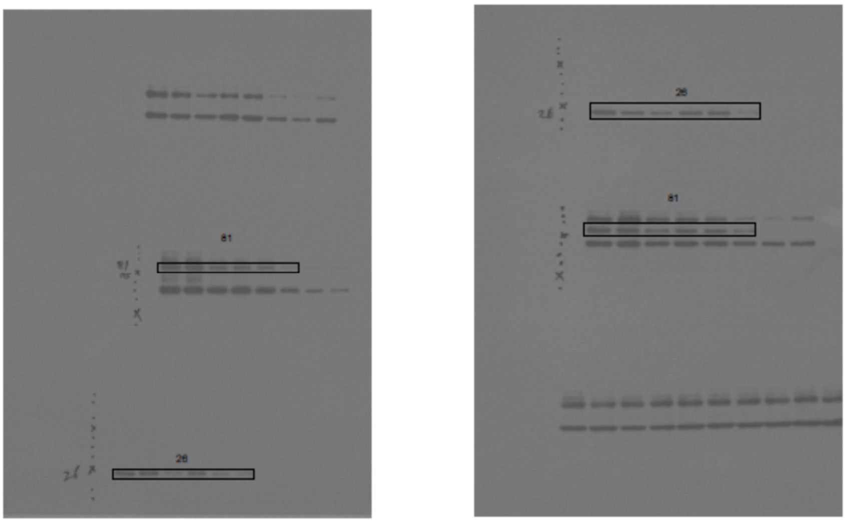

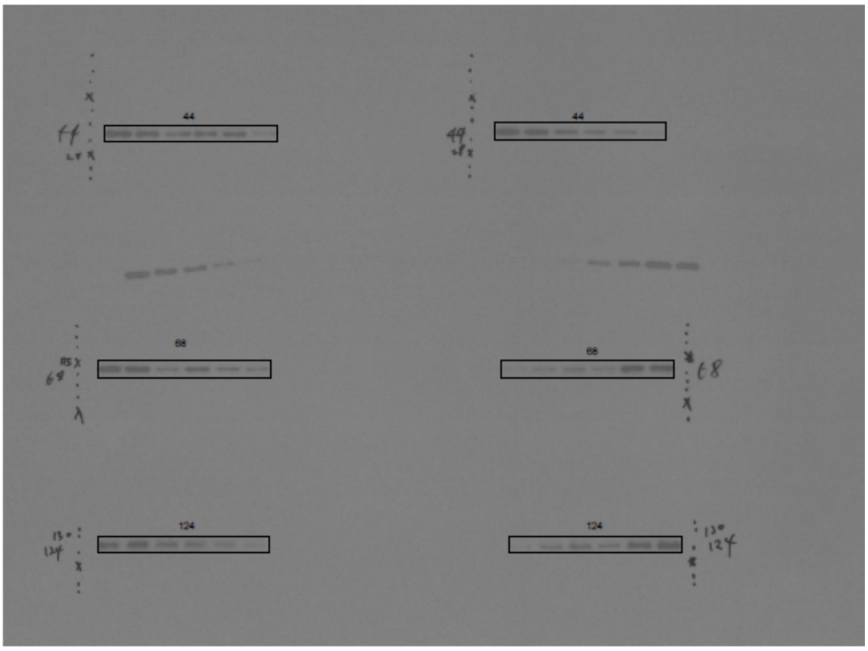
44
44
68
68
124
124

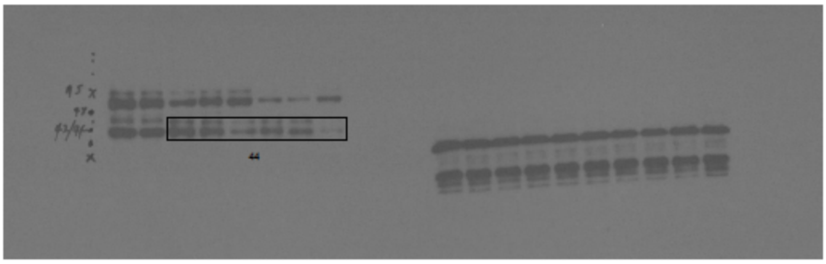
44

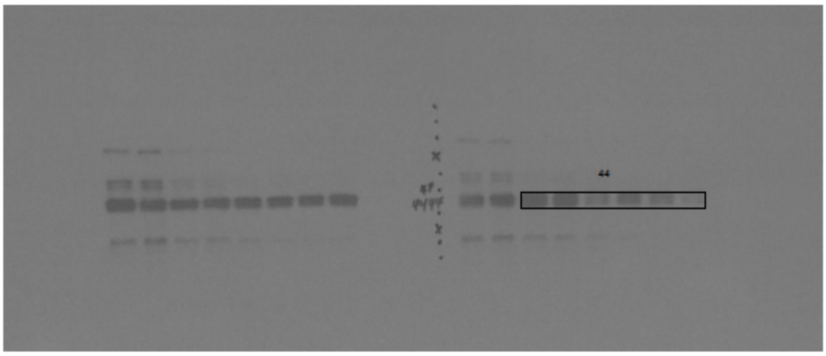
44

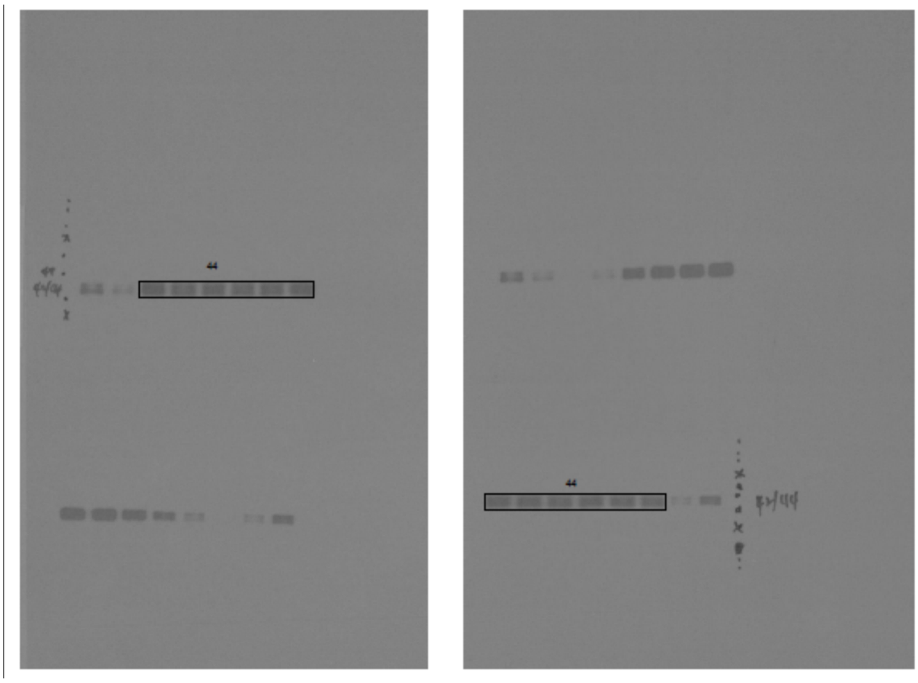

<u>Supplementary Figure 5A:</u> Immunoblot analysis with antibody- related to marker of the FGFR and EMT on the GSPY and GSPO xenograft models after treatment of a multikinase inhibitor, sorafenib and lenvatinib. Equal amounts of protein (20 μg) were separated on 8–10% sodium dodecyl sulfate-polyacrylamide gels.

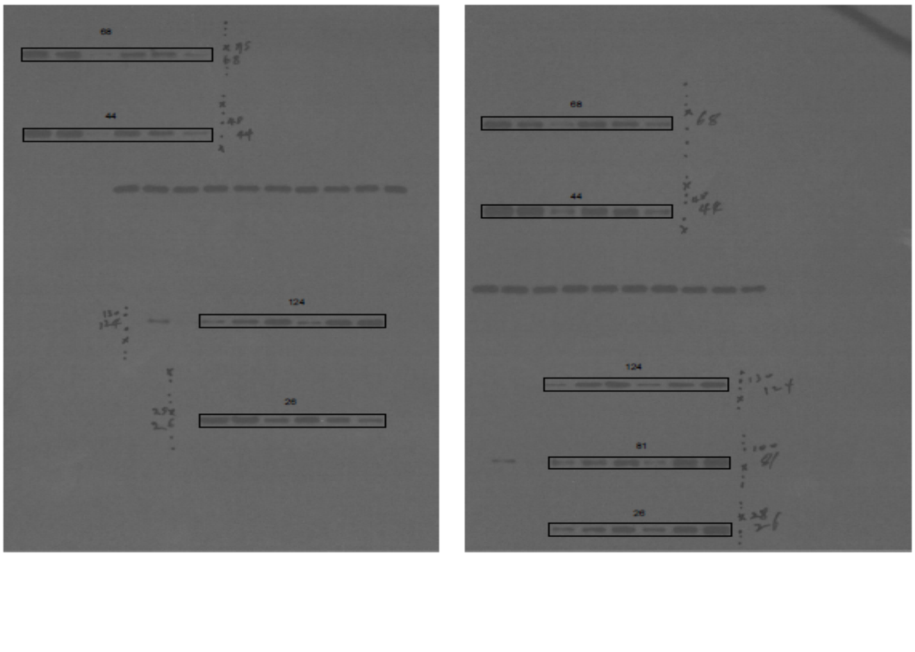

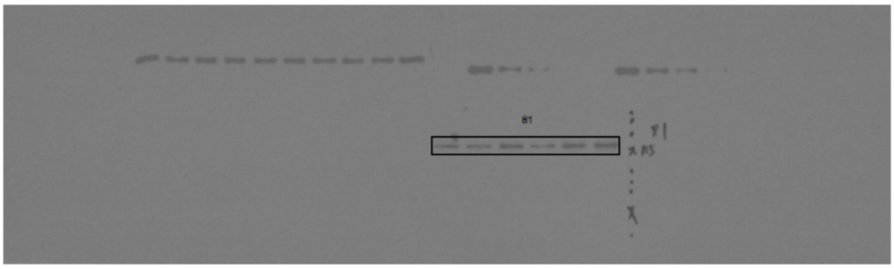
81

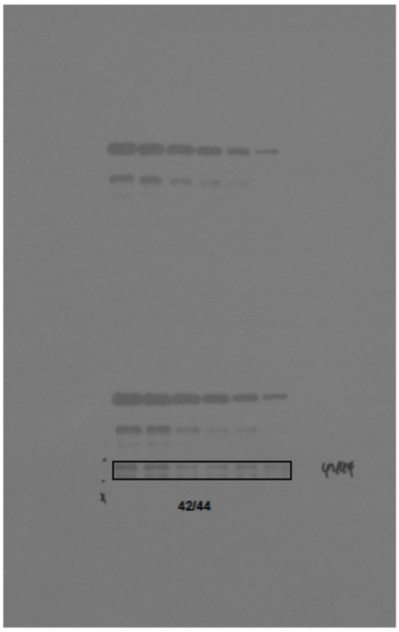
42/44

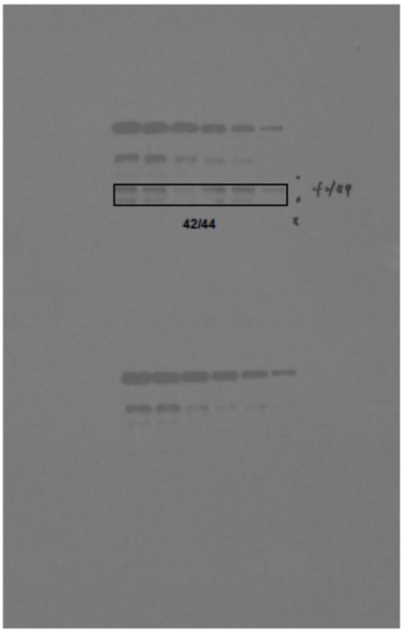
42/44
